# Using Machine Learning Algorithms to Predict Candidaemia in ICU Patients With New-Onset Systemic Inflammatory Response Syndrome

**DOI:** 10.3389/fmed.2021.720926

**Published:** 2021-08-19

**Authors:** Siyi Yuan, Yunbo Sun, Xiongjian Xiao, Yun Long, Huaiwu He

**Affiliations:** ^1^Department of Critical Care Medicine, State Key Laboratory of Complex Severe and Rare Diseases, Peking Union Medical College Hospital, Peking Union Medical College, Chinese Academy of Medical Sciences, Beijing, China; ^2^Department of Critical Care Medicine, The Affiliated Hospital of Qingdao University, Qingdao, China; ^3^Department of Critical Care Medicine, The First Affiliated Hospital of Fujian Medical University, Fuzhou, China

**Keywords:** critical care, XGBoost, predictive model, Candida, invasive fungal diseases

## Abstract

**Background:** Distinguishing ICU patients with candidaemia can help with the precise prescription of antifungal drugs to create personalized guidelines. Previous prediction models of candidaemia have primarily used traditional logistic models and had some limitations. In this study, we developed a machine learning algorithm trained to predict candidaemia in patients with new-onset systemic inflammatory response syndrome (SIRS).

**Methods:** This retrospective, observational study used clinical information collected between January 2013 and December 2017 from three hospitals. The ICU patient data were used to train 4 machine learning algorithms–XGBoost, Support Vector Machine (SVM), Random Forest (RF), ExtraTrees (ET)–and a logistic regression (LR) model to predict patients with candidaemia.

**Results:** Of the 8,002 cases of new-onset SIRS (in 7,932 patients) included in the analysis, 137 new-onset SIRS cases (in 137 patients) were blood culture positive for candidaemia. Risk factors, such as fungal colonization, diabetes, acute kidney injury, the total number of parenteral nutrition days and renal replacement therapy, were important predictors of candidaemia. The XGBoost machine learning model outperformed the other models in distinguishing patients with candidaemia [XGBoost vs. SVM vs. RF vs. ET vs. LR; area under the curve (AUC): 0.92 vs. 0.86 vs. 0.91 vs. 0.90 vs. 0.52, respectively]. The XGBoost model had a sensitivity of 84%, specificity of 89% and negative predictive value of 99.6% at the best cut-off value.

**Conclusions:** Machine learning algorithms can potentially predict candidaemia in the ICU and have better efficiency than previous models. These prediction models can be used to guide antifungal treatment for ICU patients when SIRS occurs.

## Introduction

Invasive fungal diseases (IFDs) are life-threatening infections, and their morbidity and mortality have increased in recent decades (1, 2). The most common microorganisms that cause IFDs are *Candida* species (3). Among IFDs, the incidence of candidaemia ranges between 2.4 and 15 in 100,000 individuals and has increased by 50% over the past 10 years (4–6). Approximately 45% of *Candida* bloodstream infections occur in critical care units and have become a leading cause of death among ICU patients (7). Previous studies have proven that early optimal antifungal treatment can decrease patient mortality (8–10). A definitive diagnosis of candidaemia mainly relies on blood culture (11–13), which takes time and can thus cause a delay in timely treatment of patients. Early recognition is very difficult, and the indiscriminate use of antifungal agents can cause drug resistance and increase the patient's economic burden. Therefore, we need a method to identify patients with candidaemia that can be performed faster than blood cultures.

Some predictive models for candidaemia have been proposed (14, 15), such as the *Candida* colonization index (CI) (9) and *Candida* score (CS) (16). However, most of the models used limited sample sizes because of the extremely low incidence of candidaemia (5, 6). Three predictive models (15, 17, 18) were built with large sample sizes and had a good negative predictive value of 99%, but the sensitivity and positive predictive value (PPV) were poor. When the specificity reached more than 80%, the sensitivity was only 40.5–51.4%, and the PPV varied from 4 to 9%. Previous studies tended to use traditional modeling methods, but the effectiveness of the models was insufficient.

Clinically, patients with candidaemia lack specific symptoms and signs. Systemic inflammatory response syndrome (SIRS) is often used to trigger clinicians to start anti-infection treatment. When a patient develops SIRS, clinicians will often use antibacterial drugs initially, but antifungal drugs are rarely used timely and accurately, likely causing delays in treating patients with candidaemia. Therefore, doctors must determine the probability of candidaemia when a patient presents with SIRS. Additionally, no predictive model has used SIRS as the starting point to determine the possibility that a patient has candidaemia.

Machine learning algorithms can be applied to help understand large quantities of existing data and to make predictions about new data. Previous studies have used machine learning methods to diagnose or distinguish different types of diseases (19, 20). Because of the extremely low incidence of candidaemia, the development of a prediction model requires a very large sample size and must overcome the imbalance between positive and negative results. Machine learning may provide advantages in the construction of prediction models for candidaemia among ICU patients.

Therefore, this study aimed to establish a new prediction model to determine the possibility of candidaemia in patients with SIRS with machine learning algorithms to improve the efficiency of predictive models and help with precisely prescribing antifungal drugs in the creation of personalized guidelines.

## Materials and Methods

### Study Design

This multicenter, retrospective study was performed using data from three hospitals (Peking Union Medical College Hospital, The Affiliated Hospital of Qingdao University, The First Affiliated Hospital of Fujian Medical University) obtained between January 2013 and December 2017.

Blood culture results and various influencing factors were retrospectively collected from the corresponding hospital information systems from patients who had been hospitalized in the ICU.

First, the patients' data from three hospitals were combined. Second, all the data were randomly divided into a training set and a validation set. The classic 2–8 principle was used to divide the data set: 80% for model training and 20% for model evaluation. Machine learning methods were used to train the prediction models with the data from the training set, and then the prediction models were applied to the data from the validation set to evaluate their efficiency.

### Ethics Approvals

Ethics approval was provided by the ethics committee of Peking Union Medical College Hospital. All of the data were anonymized before sharing with researchers.

### Patients

Patients who were admitted to the above target hospitals and had new-onset SIRS from 2013 to 2017 were selected as the subjects of the study. New-onset SIRS needed to meet the following criteria: (1) SIRS occurred in the ICU; (2) blood culture was obtained during the course of SIRS; (3) no previous SIRS within 24 h.

### Diagnostic Criteria

SIRS was defined when at least two of the following criteria were met (21): (1) body temperature >38°C or <36°C; (2) heart rate > 90 beats/min; (3) respiration rate > 20 times per min or hyperventilation (PaCO_2_ <32 mmHg); and (4) leukocyte count > 12 × 10^9^/L or <4 × 10^9^/L or neutrophil (rod granulocyte) percentage > 10%.

SIRS can occur many times during a single hospitalization. To avoid repeat measurement, we identified new-onset SIRS as SIRS that occurred after ICU admission and after at least 24 h of a previous SIRS event if multiple SIRS events occurred. SIRS-related candidaemia was defined if a *Candida* species was identified from blood samples collected within SIRS.

### Laboratory Tests

Two automated blood culture systems were used during the study period: a Bactec™ system (Becton Dickinson, Sparks, Maryland, USA) and a Bact/Alert®3D system (bioMérieux, Marcy l'Etoile, France).

### Data Collection and Risk Factor Definitions

We identified 28 risk factors with strong clinical significance with candidaemia by searching previous studies (see Table 1). The risk factors are mainly divided into four groups: basic patient factors, primary or combined diseases, laboratory tests, and treatment. We retrospectively collected the data involved in the research in the electronic medical record systems of the three hospitals. Colonization was defined as the presence of *Candida* species in non-significant samples taken from one or more body sites, including the oropharynx, stomach, urine, or tracheal aspirates (16). Samples were collected after ICU admission and before the collection of blood samples. Colonization information was collected based on the judgement of clinicians and clinical requirements. We retrospectively collected data about colonization from the ICU database, and not all of the patients had actively collected cultures from the oropharynx, stomach, urine, or tracheal aspirates. A previous history of fungal infection was defined as patients with invasive fungal disease before this hospitalization that was recorded in the history of past illness or reported by the patients themselves.

**Table 1:** Risk factors for previous researches.

	**Risk factors**
Basic information	Age
	Sex
	Colonization
	Length of ICU stay
	Length of hospital stay
Primary or combined diseases	Diabetes
	Acute renal injury
	History of fungal infection
	Pancreatitis
	Severe Sepsis
	Malignant tumor
	HIV/AIDS
Laboratory tests	BDG: beta-D-glucan
	NEUT^#^ ≤ 1.5 × 10^9^/L
	LYM^#^ ≤ 1 × 10^9^/L
Treatment	Total parenteral nutrition
	Mechanical ventilation
	Central venous catheter
	Abdominal surgery
	Broad-spectrum antibiotic use
	Corticosteroid therapy or immunosuppressive use
	Chemotherapy drug use
	Renal replacement therapy
	Organ transplant
	Days of total parenteral nutrition
	Days of mechanical ventilation
	Days of central venous catheter
	Days of renal replacement therapy

1,3-β-D-glucan (BDG) was defined as positive with a cut-off value of 80 pg/ml (22). The measurement occurred after ICU admission and before blood samples were collected. If there was more than one BDG result, the BDG closest to the SIRS was chosen.

### Model Training

The code of the model training part of this study is written in python (python 3.7.0). We divided the data into a training set and test set, 80% for model training, and 20% for model evaluation. We used stratified division to ensure the distribution of positive and negative cases. In order to deal with the imbalance of sample categories, the SMOTE algorithm is used in this study (the mechanism of SMOTE is listed in the Appendix). The training set was used to construct five prediction models (logistic regression model, support vector machine model, random forest model, extratree model and XGBoost model). A detailed description of the five models is provided in the Appendix. Parameter tuning is performed for each model to improve the efficiency of the models.

### Model Evaluation

The test set was used to evaluate the performance of five different models. We have used five model evaluation index, including sensitivity, specificity, positive predictive value (PPV), negative predictive value (NPV) and area under curve (AUC), to compare the performance of five models. The model with the best efficiency was chosen as the final model.

## Results

### Study Population

**Figure 1:**
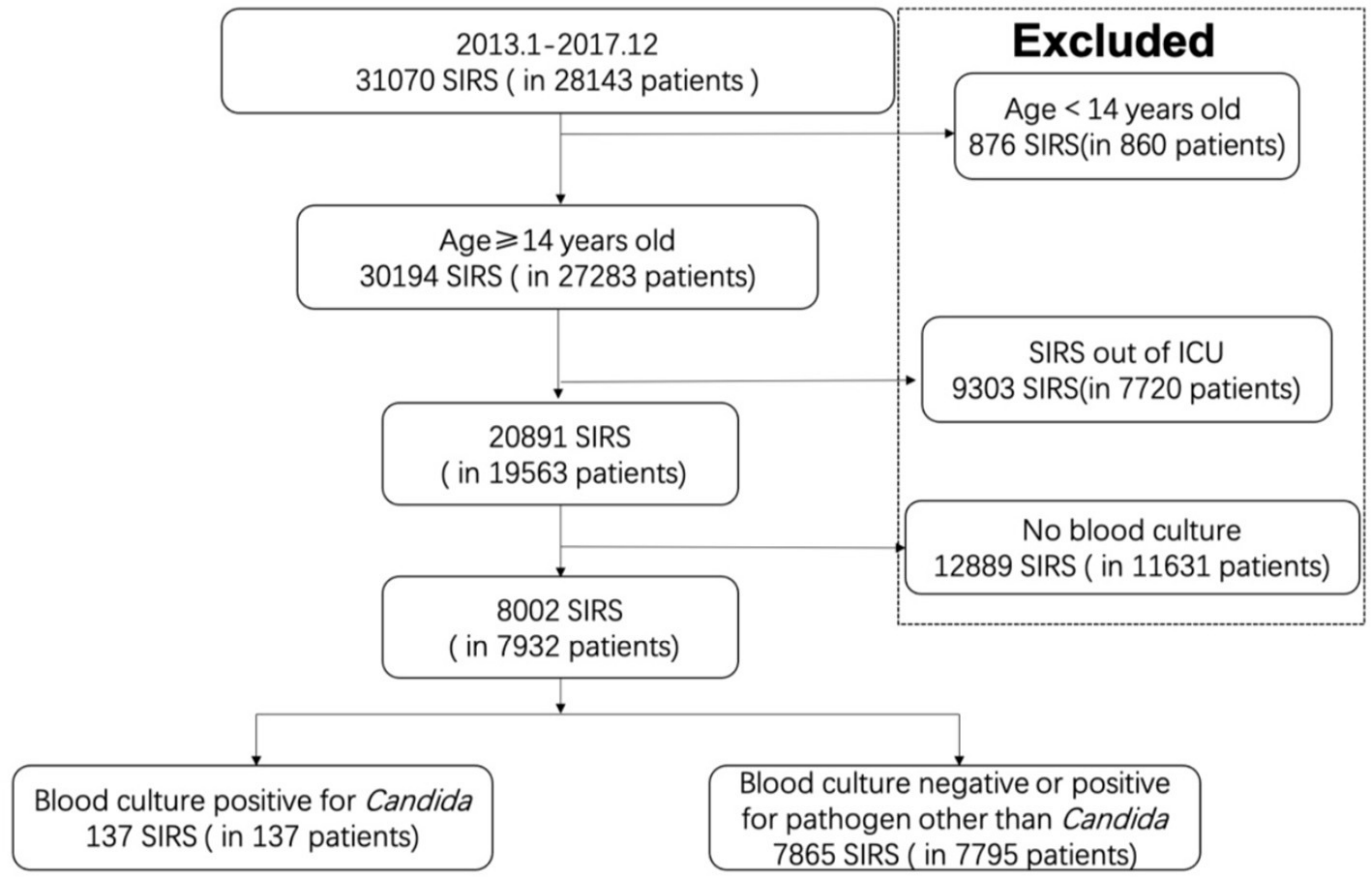
Flowchart for enrollment.

In total, 3,1070 new-onset SIRS incidents for 28,143 patients were included in this study. Excluding 876 new SIRS cases that occurred in 860 patients younger than 14 years old, 9,303 SIRS cases developed outside the ICU, and 20,891 new SIRS cases remained. Among these cases, 8,002 had corresponding blood culture results, among whom 137 were positive for *Candida* in blood culture and 7,865 were negative or were positive for a pathogen other than *Candida*. The flowchart of enrolment is described in Figure 1.

The patients were all from the ICU, the median age was 57.4 years [39.9–74.9], and 61.2% were male.

### Risk Factor Screening

We selected 28 risk factors through literature search, conducted retrospective data collection and analyzed the distribution of risk factors in different groups (Table 2).

**Table 2:** Distribution of 28 risk factors.

**Risk factor**	**Status**	**IC negative**	**IC positive**	***p* value**
Total		7,865	137	
Age, mean (SD)		57.3 (17.5)	57.64 (17.52)	0.859
Colonization (%)	No	7,346 (93.4)	93 (67.9)	<0.001
	Yes	519 (6.6)	44 (32.1)	
Length of ICU stay, mean (SD)		3.15 (5.58)	13.70 (28.28)	<0.001
Length of hospital stay, mean (SD)		7.19 (9.97)	18.32 (29.27)	<0.001
Diabetes (%)	No	6,331 (80.5)	105 (76.6)	0.308
	Yes	1,534 (19.5)	32 (23.4)	
Acute kidney injury (%)	No	4,461 (56.7)	67 (48.9)	0.081
	Yes	3,404 (43.3)	70 (51.1)	
Pancreatitis (%)	No	7,655 (97.3)	133 (97.1)	1.000
	Yes	210 (2.7)	4 (2.9)	
Malignant tumor (%)	No	6,424 (81.7)	102 (74.5)	0.040
	Yes	1,441 (18.3)	35 (25.5)	
Sepsis(%)	No	7,767 (98.8)	133 (97.1)	0.178
	Yes	98 (1.2)	4 (2.9)	
History of fungal infection(%)	No	7,859 (99.9)	134 (97.8)	<0.001
	Yes	6 (0.1)	3 (2.2)	
HIV(%)	No	7,851 (99.8)	137 (100.0)	1.000
	Yes	14 (0.2)	0 (0.0)	
BDG positive(%)	No	7,684 (97.7)	80 (58.4)	<0.001
	Yes	181 (2.3)	57 (41.6)	
NEUT^#^ ≤ 1.5 × 10^9^/L(%)	No	7,730 (98.3)	128 (93.4)	<0.001
	Yes	135 (1.7)	9 (6.6)	
Broad-spectrum antibiotic use (%)	No	905 (11.5)	6 (4.4)	0.014
	Yes	6,960 (88.5)	131 (95.6)	
Corticosteroid therapy or immunosuppressive use (%)	No	6,140 (78.1)	90 (65.7)	0.001
	Yes	1,725 (21.9)	47 (34.3)	
Chemotherapy drug use (%)	No	7,808 (99.3)	136 (99.3)	1.000
	Yes	57 (0.7)	1 (0.7)	
Total parenteral nutrition (%)	No	6,296 (80.1)	73 (53.3)	<0.001
	Yes	1,569 (19.9)	64 (46.7)	
Mechanical ventilation (%)	No	2,692 (34.2)	28 (20.4)	0.001
	Yes	5,173 (65.8)	109 (79.6)	
Central venous catheter (%)	No	2,096 (26.6)	14 (10.2)	<0.001
	Yes	5,769 (73.4)	123 (89.8)	
Renal replacement therapy (%)	No	6,823 (86.8)	93 (67.9)	<0.001
	Yes	1,042 (13.2)	44 (32.1)	
Abdominal surgery (%)	No	7,417 (94.3)	117 (85.4)	<0.001
	Yes	448 (5.7)	20 (14.6)	
Organ transplant (%)	No	7,852 (99.8)	137 (100.0)	1.000
	Yes	13 (0.2)	0 (0.0)	
Days of total parenteral nutrition mean (SD)		0.50 (1.45)	2.29 (3.60)	<0.001
Days of mechanical ventilation mean (SD)		2.23 (3.04)	5.43 (5.37)	<0.001
Days of central venous catheter mean (SD)		2.96 (3.66)	6.69 (5.73)	<0.001
Days of renal replacement therapy mean (SD)		0.38 (1.41)	1.57 (3.18)	<0.001
Sex (%)	Male	4,811 (61.2)	86 (62.8)	0.769
	Female	3,054 (38.8)	51 (37.2)	
LYM^#^ ≤ 1 × 10^9^/L (%)	No	1,608 (20.4)	8 (5.8)	<0.001
	Yes	6,257 (79.6)	129 (94.2)	

### Prediction Model Construction Using XGBoost

**Figure 2:**
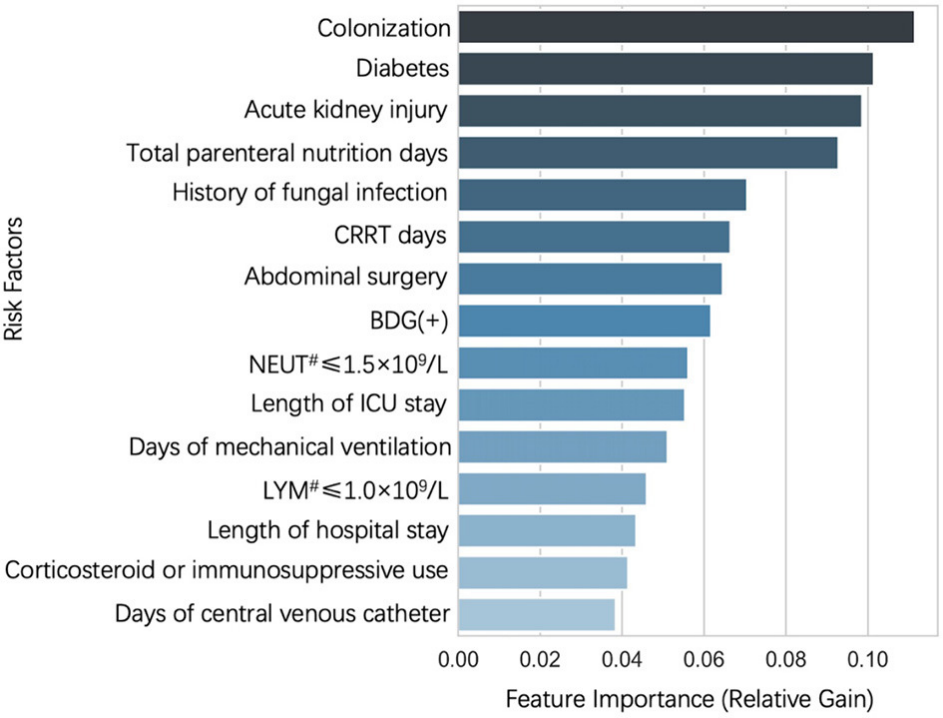
The 15 features with the highest relative gain for model predicting candidemia.

The area under the curve (AUC) for the XGBoost model ranged from 0.57 to 0.91 using different risk factors as measured by the importance score as input (Table 3). By comparing the effectiveness of models incorporating different numbers of risk factors, we chose 15 important risk factors to train the prediction models. The importance score of the 15 risk factors is shown in Figure 2.

**Table 3:** Efficiency of XGBoost model with different number of risk factors.

**Number of risk factors**	**AUC**	**Sensitivity**	**Specificity**	**PPV**
22	0.891	0.77	0.89	0.12
20	0.891	0.77	0.89	0.12
19	0.891	0.81	0.85	0.09
18	0.889	0.84	0.85	0.1
17	0.887	0.84	0.86	0.11
16	0.906	0.81	0.88	0.12
15	0.909	0.81	0.89	0.12
14	0.906	0.81	0.89	0.13
13	0.894	0.77	0.88	0.12
12	0.887	0.77	0.89	0.12
11	0.904	0.81	0.87	0.11
10	0.891	0.81	0.86	0.1
9	0.894	0.81	0.86	0.1
8	0.871	0.77	0.89	0.12
7	0.867	0.77	0.88	0.11
6	0.860	0.77	0.85	0.1
5	0.731	0.61	0.85	0.07
4	0.744	0.65	0.84	0.08
3	0.691	0.52	0.87	0.08
2	0.669	0.45	0.86	0.06
1	0.567	0	1	0

### Performance of the Models

**Figure 3:**
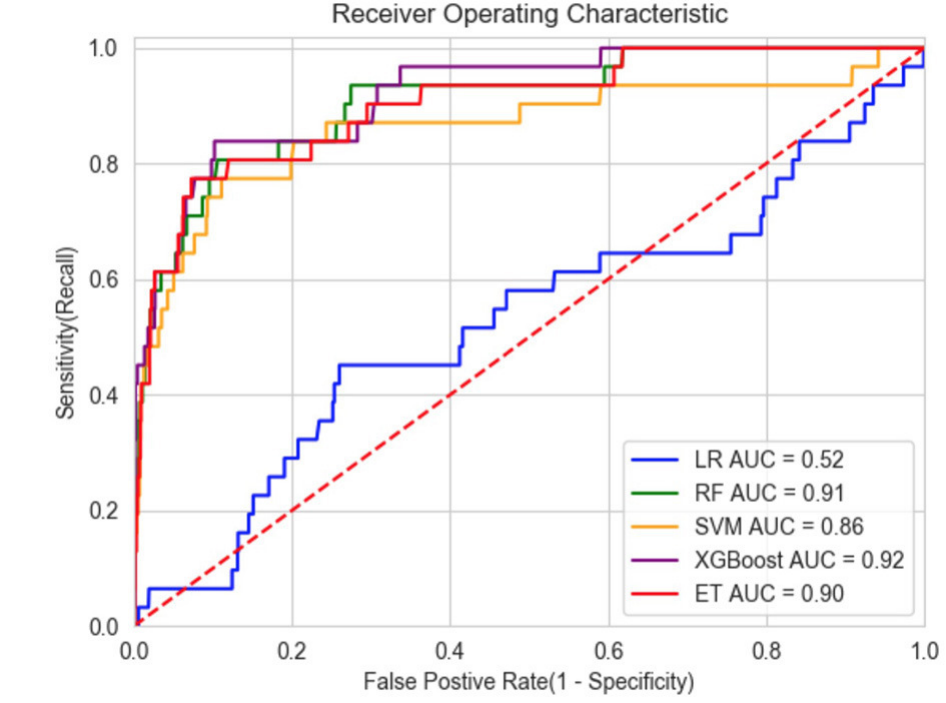
ROC of models. LR, Logistic regression; RF, Random Forest; SVM, Support Vector Machines; ET, ExtraTree.

The efficiency of the five different models is shown in Table 4, and the model receiver operating characteristic (ROC) curves are shown in Figure 3. When we set the cut-off value to 0.030, XGBoost achieved the best performance with a sensitivity of 84%, a specificity of 89% and a negative predictive value of 99.6%. Additionally, the XGBoost model achieved the best prediction performance among the machine learning models and traditional regression model.

**Table 4:** Performance of models.

**Method**	**Cut-off** **value**	**Sensitivity**	**Specificity**	**PPV**	**NPV**	**AUC**
LR	0.019	0.58	0.49	0.02	0.98	0.521 ± 0.028
RF	0.056	0.71	0.92	0.15	0.99	0.911 ± 0.015
SVM	0.016	0.87	0.64	0.05	1	0.863 ± 0.022
XGBoost	0.03	0.84	0.89	0.13	0.996	0.924 ± 0.013
ET	0.044	0.77	0.9	0.13	0.995	0.904 ± 0.014

*LR, Logistic regression; RF, Random Forest; SVM, Support Vector Machines; ET, ExtraTree*.

## Discussion

This study established a machine learning candidaemia prediction model that could be implemented in a computer program. When an ICU patient develops SIRS, real-time bedside assessment of the possibility of developing candidaemia can guide the appropriate use of antifungal drugs. To our best knowledge, this is the first machine learning-based model developed to predict candidaemia. The final model was proven to have better performance than previous prediction models. Because the machine learning model had a very high negative predictive value larger than 99%, a negative result can effectively exclude people without candidaemia, preventing the use of antifungal therapy.

### Comparison of Different Candidemia Prediction Models

Although predictive models for candidaemia have improved in the last few decades, most were trained by traditional logistic regression, and some have not been validated in large validation cohorts (8, 9).

Five well-accepted candidaemia prediction models were developed from 1994 to 2016 (9, 10, 16–18). Three of them (15, 17, 18) had a large sample size and a good negative predictive value from 99.7 to 99.9%, but the sensitivity and positive predictive value were poor. Although the specificity reached more than 80%, the model sensitivity was only 40.5–51.4%, and the PPV varied from 4–9%. Leon *et al* constructed the “Candida score”, which achieved a sensitivity of 89%, a specificity of 74% and an AUC of 0.847 (16). Another study also produced a model with good efficiency (9). However, these two models were only developed using data from patients with *Candida* colonization. Consequently, the models can only be used with restricted populations. In the present study, the XGBoost model had very high efficiency with an AUC of 0.92, a sensitivity of 84%, a specificity of 89%, and a negative predictive value of 99.6%. The PPV was not sufficiently high (13%) but was better than that of other prediction models (15, 17, 18). Because the machine learning model had a very high negative predictive value of 99.6%, a negative result can effectively exclude people without candidaemia, indicating that antifungal therapy should not be used. Because of the low number of patients with candidaemia in this study, the positive predictive value was not sufficiently high. A positive result would indicate a probability of the patient developing candidaemia of 13%, which still substantially increases the probability of the effective use of antifungal drugs. Our model can be combined with other prediction methods with high positive predictive value to conduct a second evaluation of patients who are positive according to the machine learning model to further improve the detection efficiency.

### Machine Learning Models in China

Because of the low incidence of candidaemia, previous prospective studies lacked a large sample size and demonstrated an imbalance between positive and negative samples. The FIRE study in the UK was a multicenter prospective study on invasive fungal disease and included 60,778 admissions from 96 critical care units (18). Although the study yielded good results, it required considerable economic and labor costs. The use of a database to establish machine learning models not only reduces the economic cost of research but also improves the effectiveness of the resulting predictive models. The validation cohort proved that the XGBoost model could achieve the best prediction performance among the different machine learning models and traditional regression models with an AUC of 0.92.

### SIRS as a Starting Point

In clinical practice, the presence of SIRS in ICU patients often leads to suspected infection. SIRS meets clinical needs and has high clinical operability as the starting point to guide antifungal therapy. Additionally, the incidence of SIRS in ICU patients is >80% (23); thus, the proposed prediction model should apply to a wide range of individuals. The innovative use of SIRS as a trigger point to create a candidaemia prediction model, combined with machine learning algorithms, will maximize the use of ICU big data and improve the immediacy and accuracy of prediction.

### Useful Software for Clinical Practice

Because this study used a machine learning method to establish the candidaemia predictive model, the test results cannot be determined simply by the weighted scores of the risk factors but must be calculated using a program. When an ICU patient becomes afflicted with SIRS, the clinician can input the corresponding risk factor values into the program, which can automatically output a positive or negative prediction result, thereby achieving real-time prediction at the bedside.

### Risk Factors Related to Candidaemia

The most important risk factors in this predictive model included fungal colonization, diabetes, acute kidney injury, total parenteral nutrition and renal replacement therapy, which are consistent with previous studies (8, 24–27). However, some risk factors mentioned in previous studies were not included in our prediction model, such as the APACHE II score (9, 28) and severe sepsis (16).

### Limitations

First, to ensure the accuracy of the study, we excluded SIRS patients without blood samples and only enrolled new-onset SIRS patients with blood cultures obtained during the course of SIRS. We acknowledge that the exclusion of the 12,894 SIRS without blood samples may introduce biases and influence the performance of the prediction model. However, the data of 8,002 SIRS for analysis were relatively large in the prediction model. Additionally, the incidence of candidaemia in all SIRS patients was approximately 0.65% (137/20,891), which was similar to that in previous studies (0.15–0.65%) (29, 30). Second, blood cultures were not obtained for 12,894 patients with SIRS. In clinical practice, the presence of SIRS in ICU patients often leads to suspected infection. However, SIRS is not the only indicator to trigger blood sample culture in clinical practice. Individual differences exist in the standard and clinical practice of blood culture. Hence, it was reasonable to observe an SIRS rate >50% without blood sample culture in the present study. Third, the study population only comprised ICU patients. Therefore, the results may not be generalizable to non-ICU patients. Fourth, the number of positive samples included in this study was relatively small because of the extremely low incidence of candidaemia, possibly affecting the effectiveness of the prediction model. Therefore, we used the SMOTE mechanism to improve the imbalance of positive and negative samples and improve the efficiency of the model. Fifth, including patients from three hospitals may have increased the bias between the hospitals. By adopting strict and consistent risk factor evaluation standards, this bias could be reduced, and the multicenter nature of the research can improve sample representativeness. Sixth, some of the risk factors did not demonstrate significant differences because of their low incidence, such as chemotherapy drugs. These risk factors are less common in the overall ICU population; therefore, their importance is difficult to judge. Additionally, the data concerning colonization were collected retrospectively, possibly influencing the accuracy of this risk factor and efficiency of the model. In the present study, the negative predictive value of BDG was high, partly because of the low incidence of candidaemia. The high negative predictive value will partly contribute to the good efficiency of the prediction model with an NPV of 99.6%. Finally, retrospective studies have inherent data biases. Although the ICU database can ensure some measure of accuracy, the efficiency of the prediction model must be further evaluated in the future.

### Conclusion

The machine learning prediction model for candidaemia has good efficiency and can guide antifungal treatment in ICU patients when new-onset SIRS occurs.

## Take-Home Message

Approximately 45% of *Candida* bloodstream infections occur in critical care units and have become a leading cause of death among ICU patients. Previous prediction models of candidaemia mostly used traditional logistic models and had some limitations. In this study, we developed a machine learning algorithm trained in predicting candidaemia in patients with new-onset systemic inflammatory response syndrome (SIRS) with good performance.

## Data Availability Statement

The datasets presented in this article are not readily available because to protect patients' privacy. Requests to access the datasets should be directed to yuansiyizqh@163.com.

## Ethics Statement

Ethics approval was provided by the ethics committee of Peking Union Medical College Hospital. All of the data were anonymized before sharing with researchers.

## Author Contributions

SY performed the experiments and statistical analysis and wrote the manuscript. YS and XX participated in the design of the study and statistical analysis. HH participated in the design of the study and helped to draft the manuscript. YL conceived of the study, participated in its design and helped to draft, and revise the manuscript. All authors have read and approved the final manuscript.

## Conflict of Interest

The authors declare that the research was conducted in the absence of any commercial or financial relationships that could be construed as a potential conflict of interest.

## Publisher's Note

All claims expressed in this article are solely those of the authors and do not necessarily represent those of their affiliated organizations, or those of the publisher, the editors and the reviewers. Any product that may be evaluated in this article, or claim that may be made by its manufacturer, is not guaranteed or endorsed by the publisher.
