# Supplementary material for: Using Machine Learning Algorithms to Predict Candidaemia in ICU Patients With New-Onset Systemic Inflammatory Response Syndrome
Source: Front Med (Lausanne). 2021 Aug 19;8:720926. doi: 10.3389/fmed.2021.720926 (PMC8416760; doi:10.3389/fmed.2021.720926)
Supplement: Supplementary file 1 [file Data_Sheet_1.docx]

**Supplementary data**

**XGBoost Method**

We review gradient tree boosting algorithms in this section. The derivation follows from the same idea in existing literatures in gradient boosting.

For a given data set with n examples and m features D = {(xi,yi)} (|D| = n,xi ∈ Rm,yi ∈ R), a tree ensemble model (shown in Fig. 1) uses K additive functions to predict the output.


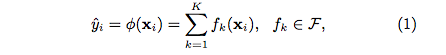


where F={f(x)=wq(x)}(q:Rm →T,w∈RT) is the space of regression trees (also known as CART). Here q represents the structure of each tree that maps an example to the corresponding leaf index. T is the number of leaves in the tree. Each fk corresponds to an independent tree structure q and leaf weights w. Unlike decision trees, each regression tree contains a continuous score on each of the leaf, we use wi to represent score on i-th leaf.


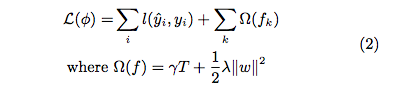


Here l is a differentiable convex loss function that measures the difference between the prediction yˆi and the target yi. The second term Ω penalizes the complexity of the model (i.e., the regression tree functions). The additional regularization term helps to smooth the final learnt weights to avoid over-fitting.

Eq.(2) includes functions as parameters and cannot be optimized using traditional optimization methods in Euclidean space. Instead, the model is trained in an additive manner. Formally, let yˆ(t) be the prediction of the i-th instance at the t-th iteration, we will need to add ft to minimize the following objective.


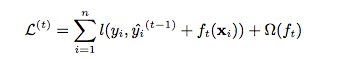
（2.1）

This means we greedily add the ft that most improves our model according to Eq. (2). Second-order approximation can be used to quickly optimize the objective in the general setting.


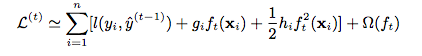
（2.2）


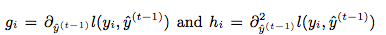


where gi and h_i_ are first and second order gradient statistics on the loss function. We can remove the constant terms to obtain the following simplified objective at step t.


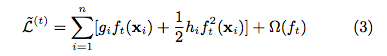


Define Ij = {i|q(xi) = j} as the instance set of leaf j. We can rewrite Eq (3) by expanding Ω as follows


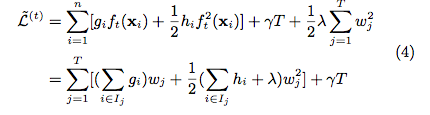


For a fixed structure q(x), we can compute the optimal weight wj∗ of leaf j by


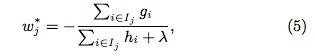


and calculate the corresponding optimal value by


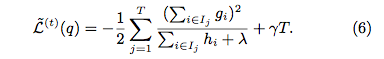


Eq (6) can be used as a scoring function to measure the quality of a tree structure q. This score is like the impurity score for evaluating decision trees, except that it is derived for a wider range of objective functions.

Normally it is impossible to enumerate all the possible tree structures q. A greedy algorithm that starts from a single leaf and iteratively adds branches to the tree is used instead. Assume that IL and IR are the instance sets of left and right nodes after the split. Lettting I = IL ∪ IR, then the loss reduction after the split is given by


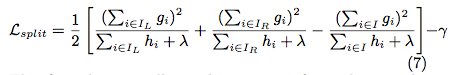


This formula is usually used in practice for evaluating the split candidates.

Support Vector Machine (SVM)

Support Vector Machine (SVM) is a widely used supervised machine learning method for binary classification which uses the training data to build a model for classification. The SVM then uses this model to classify, using attribute data, each instance in the test set.

The main concept behind linear SVMs is to maximize the distance between two parallel boundaries or hyperplanes which are defined by support vectors. The objective is to construct a separating hyperplane which achieves maximum separation between the 2 classes.

When generating the model for classification, SVM looks for the maximum margin hyperplane which divides the training data into two categories.

SVM uses the coordinates of the nearest training data points in both classes in order to create the largest possible separation between border values in each class.


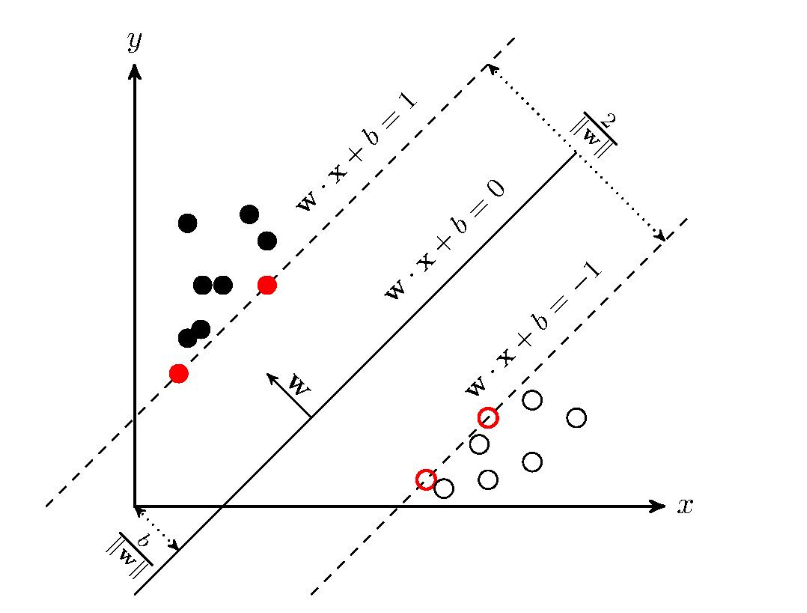


Random Forest，RF

Random forest was proposed by Breiman. In addition to constructing each tree using a different bootstrap sample of the data, random forests change how the classiﬁcation or regression trees are constructed. In standard trees, each node is split using the best split among all variables. In a random forest, each node is split using the best among a sub-set of predictors randomly chosen at that node. This somewhat counterintuitive strategy turns out to per-form very well compared to many other classiﬁers, including discriminant analysis, support vector ma-chines and neural networks, and is robust against overﬁtting (Breiman, 2001). In addition, it is very user-friendly in the sense that it has only two parameters (the number of variables in the random subset at each node and the number of trees in the forest), and is usually not very sensitive to their values.

The randomForest package provides an R interface to the Fortran programs by Breiman and Cutler (available at http://www.stat.berkeley.edu/users/breiman/).

ExtraTree，ET

In the extreme case, it builds totally randomized trees whose structures are independent of the output values of the learning sample. The strength of the randomization can be tuned to problem speciﬁcs by the appropriate choice of a parameter. We evaluate the robustness of the default choice of this parameter, and we also provide insight on how to adjust it in particular situations. Besides accuracy, the main strength of the resulting algorithm is computational efﬁciency. A bias/variance analysis of the Extra-Trees algorithm is also provided as well as a geometrical and a kernel characterization of the models induced.

Synthetic minority oversampling Technology, SMOTE

SMOTE is an improved scheme based on random oversampling algorithm. Because random oversampling adopts the strategy of simply copying samples to increase a few class samples, it is easy to produce the problem of model over fitting. Even if the information learned from the model is too specific to be generalized, the basic idea of smote algorithm is to synthesize new minority class samples In this paper, the synthesis strategy is: for a minority sample in the data, k-nearest neighbors (KNN) algorithm is used to find the nearest K minority samples. Where k needs to be specified artificially, and the definition of distance is Euclidean distance of feature space. Then, one of the k nearest neighbor points is randomly selected. Finally, the newly generated sample points are located on the line connecting the randomly selected nearest neighbor points with the original ones. Smote algorithm is to interpolate the minority samples, and the adaptive composite sampling algorithm is to automatically determine the number of composite samples generated by the minority samples without affecting the original distribution of the data as much as possible.
